# Recurrent neural network for trajectory tracking control of manipulator with unknown mass matrix

**DOI:** 10.3389/fnbot.2024.1451924

**Published:** 2024-08-19

**Authors:** Jian Li, Junming Su, Weilin Yu, Xuping Mao, Zipeng Liu, Haitao Fu

**Affiliations:** College of Information Technology, Jilin Agricultural University, Changchun, China

**Keywords:** recurrent neural network (RNN), trajectory tracking, manipulator control, dynamic model, unknown mass matrix

## Abstract

Real-world robotic operations often face uncertainties that can impede accurate control of manipulators. This study proposes a recurrent neural network (RNN) combining kinematic and dynamic models to address this issue. Assuming an unknown mass matrix, the proposed method enables effective trajectory tracking for manipulators. In detail, a kinematic controller is designed to determine the desired joint acceleration for a given task with error feedback. Subsequently, integrated with the kinematics controller, the RNN is proposed to combine the robot's dynamic model and a mass matrix estimator. This integration allows the manipulator system to handle uncertainties and synchronously achieve trajectory tracking effectively. Theoretical analysis demonstrates the learning and control capabilities of the RNN. Simulative experiments conducted on a Franka Emika Panda manipulator, and comparisons validate the effectiveness and superiority of the proposed method.

## 1 Introduction

With the rapid development of modern robot research and development technology, manipulators have permeated various aspects of human life, such as space explorations (Ma et al., 2023a) and smart factories (Abate et al., 2022). Its fundamental functionality lies in trajectory tracking, where specific tasks are accomplished by executing predefined end-effector trajectories (Jin et al., 2024b). This involves the control of robot kinematics and dynamics (Liao et al., 2022; Lian et al., 2024; Sun et al., 2024). To exert control over the robot, desired joint attributes should be obtained according to the task trajectory and converted into the corresponding joint torques (Müller et al., 2023). Numerous algorithms, such as pseudoinverse methods (Guo et al., 2018; Sun et al., 2023a) and model predictive control method (Jin et al., 2023), have been designed to achieve precise control of the manipulator. However, these algorithms rely on accurate robot models and struggle to control the robot effectively when its parameters change. In practical applications, it is common for robot model parameters to vary, especially when robots are modified to perform different tasks in diverse application scenarios (Xiao et al., 2022; Xie and Jin, 2024). Reliable model-free control methods need to be designed to enable effective control of robots after the parameter changes.

In recent decades, there are many emerging algorithms to address the control issues of manipulators (Liao et al., 2024; Yan et al., 2024), which are considered from the velocity level (Zhang et al., 2019; Sun et al., 2022), acceleration level (Wen and Xie, 2024), or torque level (Hua et al., 2023). For instance, to eliminate the joint-angle drift and prevent excessive joint velocity, a velocity-level bi-criteria optimization scheme is provided for coordinated path tracking of manipulators, focusing on the velocity aspect (Xiao et al., 2017). Additionally, a data-driven acceleration-level scheme is introduced to address control continuity and stability issues for manipulator (Wen and Xie, 2024). However, most of these studies focus solely on kinematics, neglecting dynamic factors (Tang and Zhang, 2022). Robot kinematics and dynamics are two fundamental domains within the field of robotics. Robot kinematics focuses on the study of the motion capabilities of robots in space, encompassing aspects such as joint angles, positions, velocities, and accelerations, without considering the effects of forces (Xie et al., 2023). In addition, robot dynamics is concerned with the impact of forces and torques on the motion of the manipulator, including the interactions between the robot and its environment. In the control of robotic manipulators, considering dynamic factors can help precisely predict the actual motion trajectory of the manipulator under various load and motion conditions, thereby improving the overall motion accuracy (Sun et al., 2023b; Xiao et al., 2023). It can also compensate for the oscillation and coupling effects in joint motion, making the movement of the manipulator smooth and stable. Furthermore, the dynamics-based studies aid in selecting the optimal drive scheme, reducing energy consumption, and enhancing energy utilization efficiency. However, manipulators frequently encounter issues with dynamic uncertainties due to the diversity of robotic grippers and uncertainties in load (Bruder et al., 2021; Liu et al., 2024b). Specifically, surgical manipulators may be equipped with different end-effectors to meet various task requirements, implying changes in dynamic parameters (Liu et al., 2024b). Moreover, in robotic-grasping tasks, unknown loads also lead to variations in robot dynamic parameters (Bruder et al., 2021). Dynamic uncertainties significantly impede the accurate control of manipulators, highlighting its research significance.

Recurrent neural networks (RNNs) have emerged as effective robot control algorithms in recent years (Liao et al., 2023; Ma et al., 2023b; Jin et al., 2024a). RNN is utilized to establish a scheme for addressing the coordination problem for multirobot systems (Cao et al., 2023; Liu et al., 2024a). In addition, RNN can mitigate uncertainties in the robot systems by enabling online learning of robot parameters (Xie et al., 2022). However, further research is needed to explore the integration of synchronous dynamic parameter learning with the kinematic model to achieve accurate trajectory tracking (Tang et al., 2024). To this aim, this study assumes the presence of deviations in the robot dynamic model and proposes an RNN for the model-free control of manipulators. Specifically, relevant control algorithms are designed at both the kinematic and dynamic levels, and an estimator of the mass matrix is proposed to compensate for the uncertainty of the dynamic model. Further verifications are carried out on a Franka Emika Panda manipulator to perform a trajectory-tracking task, taking into account dynamic uncertainties. In addition, compared with the existing methods, the superiorities of the proposed RNN lie in the following two aspects:

- Compared with kinematics-based methods (Guo et al., 2018; Jin et al., 2023, 2024b), the proposed RNN bridges the robot kinematics and robot dynamics models through joint acceleration signals, considering the motion feature and the dynamic behavior of manipulators.
- Compared with dynamics-based methods (Shojaei et al., 2021; Zong and Emami, 2021), the proposed RNN addresses the dynamic uncertainty problem by estimating the mass matrix online and realizes synchronous trajectory tracking.

Through the introduction of the above basic content, the specific research of this study is organized as follows. Section 2 explains the kinematic relationship between the joint angle of the manipulator and the end-effector. In Section 3, a corresponding RNN is designed. Subsequently, the learning and control ability of the proposed RNN are analyzed theoretically in Section 4. Finally, simulations and comparisons are carried out in Section 5.

## 2 Kinematic controller

The forward kinematics of the manipulator describes the mapping relationship between the joint angle and the end-effector position, described as *f*(***q***) = ***r***, where ***q*** ∈ ℝ^*a*^ is the joint angle, ***r*** ∈ ℝ^*b*^ denotes the position of the end-effector, and *f*(·) stands for the non-linear mapping. Furthermore, the time derivative of the forward kinematics is derived as


(1) $$\begin{array}{l} L\overset{\cdot }{q}=\overset{\cdot }{r}, \end{array}$$

where ***L*** = ∂*f*(***q***)/∂***q*** ∈ ℝ^*b*×*a*^ is the Jacobian matrix, $\overset{\cdot }{q}$ denotes the joint velocity, and $\overset{\cdot }{r}$ is the velocity of the end-effector. Concerning the joint acceleration level, taking the time derivative of Equation 1 leads to


(2) $$\begin{array}{l} \overset{\cdot }{L}\overset{\cdot }{q}+L\ddot{q}=\ddot{r}, \end{array}$$

where $\overset{\cdot }{L}$ is the time derivative of ***L***, $\ddot{q}$ represents the joint acceleration, and $\ddot{r}$ denotes the acceleration of the end-effector. Building upon Equation 2, the desired joint acceleration can be obtained by the following kinematic controller:


(3) $$\begin{array}{l} \ddot{q}=L^{†}\left({\ddot{r}}_{\text{d}}-\overset{\cdot }{L}\overset{\cdot }{q}-v\right), \end{array}$$

where $v=\beta \left(\overset{\cdot }{r}-{\overset{\cdot }{r}}_{\text{d}}\right)+\zeta \left(r-r_{\text{d}}\right)$ denotes the error feedback term; ***r***_d_, ${\overset{\cdot }{r}}_{\text{d}}$, and ${\ddot{r}}_{\text{d}}$ are the desired position, velocity, and acceleration of the trajectory tracking task; superscript ^†^ denotes the pseudoinverse operation of a matrix with ***L***^†^ = ***L***^T^(***LL***^T^)^−1^; and β>0 and α>0 are convergence coefficients. On the one hand, kinematic controller (Equation 3) utilizes the minimization function of the pseudoinverse operation to obtain the desired joint acceleration (Wen and Xie, 2024). On the other hand, it takes the desired trajectory tracking task as input and incorporates feedback of the tracking error, leading to improved trajectory tracking performance. In addition, kinematic controller (Equation 3) can apply traditional methods to avoid the singularity issues, such as the damped least squares method (Xie et al., 2024). Specifically, a damped term can be added in the computation of the pseudoinverse. The specific calculation formula is ***L***^T^(***LL***^T^ + *μ**I***)^−1^, where μ>0 denotes a tiny parameter and ***I*** denotes the identity matrix. By doing so, the infinite values caused by zero eigenvalues in the pseudoinverse operation can be avoided.

## 3 Recurrent neural network design

**Figure 1:**
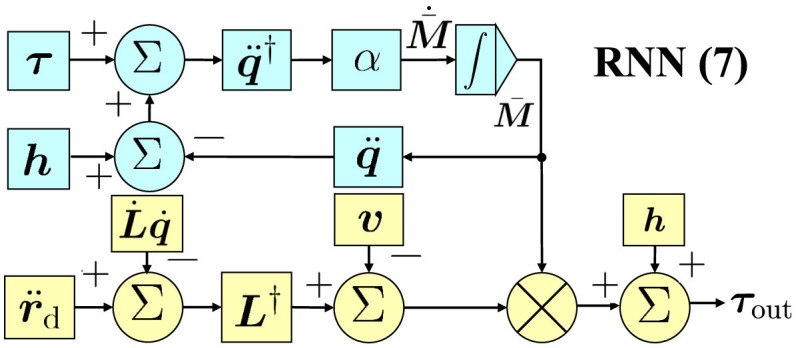
A control flow chart of RNN (Equation 7).

Robot dynamics refers to the mathematical description of the relationship between joint torques, dynamic parameters, and joint motions in a robotic system. Specifically, the dynamic model of a manipulator can be written as


(4) $$\begin{array}{l} \tau =M\left(q\right)\ddot{q}+c\left(q,\overset{\cdot }{q}\right)+g\left(q\right), \end{array}$$

where ***τ*** ∈ ℝ^*a*^ represents the joint torque, ***M***(***q***) ∈ ℝ^*a*×*a*^ is the mass matrix, $c\left(q,\overset{\cdot }{q}\right)\in {\mathbb{R}}^{a}$ is the Coriolis and centrifugal vector, and ***g***(***q***) ∈ ℝ^*a*^ denotes the gravity vector. Generally, traditional methods, such as Guo et al. (2018), are capable of performing accurate dynamic control by relying on precise dynamic models (Equation 4). However, in real-world applications, it is common for manipulators to undergo modifications to perform various tasks, resulting in changes in their dynamic parameters. Given the assumption that the change occurs in the inertia matrix, we design an estimated inertia matrix $\bar{M}\in {\mathbb{R}}^{a\times a}$ to effectively mitigate dynamic uncertainties. As a result, the following state equation is established:


(5) $$\begin{array}{l} \bar{\tau }=\bar{M}\left(q\right)\ddot{q}+c\left(q,\overset{\cdot }{q}\right)+g\left(q\right), \end{array}$$

where $\bar{\tau }\in {\mathbb{R}}^{a}$ is the corresponding joint torque. When the estimated inertia matrix converges to the actual one, it indicates that the dynamic uncertainty issue is solved. To this aim, an estimation equation is presented as follows:


(6) $$\begin{array}{l} \overset{\cdot }{\bar{M}}=\alpha \left(\tau -\bar{\tau }\right){\ddot{q}}^{†}, \end{array}$$

where $\overset{\cdot }{\bar{M}}$ determines the evolution direction of $\bar{M}$, and α > 0 stands for the convergence coefficient. In Equation 6, ***τ*** is measured in real time. Combining Equations 3, 5, 6, an RNN is designed as follows:


(7a) $$\begin{array}{l} \overset{\cdot }{\bar{M}}=\alpha \left(\tau -\bar{M}\left(q\right)\ddot{q}+h\left(q,\overset{\cdot }{q}\right)\right){\ddot{q}}^{†}, \end{array}$$

(7b) $$\begin{array}{l} \tau_{\text{out}}=\bar{M}\left(q\right)\left(L^{†}\left({\ddot{r}}_{\text{d}}-\overset{\cdot }{L}\overset{\cdot }{q}\right)-v\right)+h\left(q,\overset{\cdot }{q}\right), \end{array}$$

where ***τ***_out_ is the output signal and $h\left(q,\overset{\cdot }{q}\right)=c\left(q,\overset{\cdot }{q}\right)+g\left(q\right)$. In addition, a control flow chart of RNN (Equation 7) is shown in Figure 1. Notably, the joint acceleration generated by Equation 3 in a kinematic manner serves as the input for Equation 7b. Furthermore, Equation 7a utilizes measurement data τ to estimate the mass matrix, which, in turn, facilitates the precise control of Equation 7b. In this context, RNN (Equation 7) demonstrates its capability to learn the mass matrix and achieve synchronous trajectory tracking via the joint torque. The parameters in RNN (Equation 7) include α, β, and ζ, which are determined through trial and error methods.

In RNN (Equation 7), we first apply kinematic controller (Equation 3) to output the joint acceleration corresponding to the trajectory task. This process belongs to the inverse kinematics solution. Subsequently, we further obtain the joint torque by calculating the obtained joint acceleration. This process belongs to the inverse dynamics of solution. Finally, the output joint torque can directly control the manipulator to perform the given task. In this control mode, robot kinematics and dynamics are combined together with joint acceleration to form a bridge.

## 4 Theoretical analysis

The following theorem provides a verification of the learning and control capabilities of the proposed RNN (Equation 7).

*Theorems:* Assuming a sufficiently large value of α, the estimated error $M-\bar{M}$ generated by Equation 7a is global convergent to a zero matrix. Based on the estimated mass matrix, Equation 7b enables accurate trajectory-tracking control of the manipulator with an unknown mass matrix.

*Proof:* By incorporating Equations 2, 3, we can rewrite Equation 7b as $\overset{\cdot }{\bar{M}}=\alpha \left(M-\bar{M}\right)\ddot{q}{\ddot{q}}^{†}$. Multiplying both sides of the equation by $\ddot{q}$ yields $\overset{\cdot }{\bar{M}}\ddot{q}=\alpha \left(M-\bar{M}\right)\ddot{q}$. Then, it follows that $\overset{\cdot }{\bar{M}}=\alpha \left(M-\bar{M}\right)$. Define an estimated error as $e=\left(M_{i}-{\bar{M}}_{i}\right)$ with *M*_*i*_ and ${\bar{M}}_{i}$ being the *i*-th column of *M* and $\bar{M}$ (*i* = 1, ⋯ , *a*), respectively. Set a Lyapunov function as $V={\left(M_{i}-{\bar{M}}_{i}\right)}^{\text{T}}\left(M_{i}-{\bar{M}}_{i}\right)$, and then, its time derivative is calculated as follows:


(8) $$\begin{array}{l} \overset{\cdot }{V}={\left(M_{i}-{\bar{M}}_{i}\right)}^{\text{T}}{\overset{\cdot }{M}}_{i}-\alpha {\left(M_{i}-{\bar{M}}_{i}\right)}^{\text{T}}\left(M_{i}-{\bar{M}}_{i}\right)\\ \;=e^{\text{T}}{\overset{\cdot }{M}}_{i}-\alpha e^{\text{T}}e\\ \;\leq ||e|{|}_{2}||{\overset{\cdot }{M}}_{i}|{|}_{2}-\alpha ||e|{|}_{2}^{2}\\ \;=||e|{|}_{2}\left(||{\overset{\cdot }{M}}_{i}|{|}_{2}-\alpha ||e|{|}_{2}\right), \end{array}$$

with ||·||_2_ being the Euclidean norm of a vector. The above equation leads to three different situations as follows:

- Situation i: $||e|{|}_{2}>||{\overset{\cdot }{M}}_{i}|{|}_{2}/\alpha$. This situation contributes to $\overset{\cdot }{V}<0$ and *V*>0, which implies that ||*e*||_2_ is convergent until $||e|{|}_{2}=||{\overset{\cdot }{M}}_{i}|{|}_{2}$.
- Situation ii: $||e|{|}_{2}=||{\overset{\cdot }{M}}_{i}|{|}_{2}/\alpha$. It leads to $\overset{\cdot }{V}\leq 0$. This suggests that ||*e*||_2_ converges to zero or maintains at $||e|{|}_{2}=||{\overset{\cdot }{M}}_{i}|{|}_{2}$.
- Situation iii: $||e|{|}_{2}<||{\overset{\cdot }{M}}_{i}|{|}_{2}/\alpha$. We deduce that $\overset{\cdot }{V}>0$ or $\overset{\cdot }{V}\leq 0$. Subsequently, it can be inferred that ||*e*||_2_ continues to increase until it reaches Situation ii or it remains unchanged or convergent.

Considering the above three situations, it can be obtained that $||e|{|}_{2}\leq ||{\overset{\cdot }{M}}_{i}|{|}_{2}/\alpha$ when *t* → ∞. Provided a sufficiently large value of α, we have that ||*e*||_2_ reaches zero when *t* → ∞. In conclusion, the estimated error $M-\bar{M}$ generated by Equation 7b globally converges to a zero matrix. Hence, applying LaSalle's invariance principle (K.Khalil, 2001), we replace $\bar{M}$ with *M* in Equation 7b and deduce


(9) $$\begin{array}{l} \tau_{\text{out}}=M\left(q\right)\left(L^{†}\left({\ddot{r}}_{\text{d}}-\overset{\cdot }{L}\overset{\cdot }{q}\right)-v\right)+h\left(q,\overset{\cdot }{q}\right). \end{array}$$

Therefore, Equation 7b enables dynamic control of the manipulator depending on the desired joint acceleration in kinematic controller (Equation 3).

The desired joint acceleration allows the manipulator to precisely follow a given trajectory, which is proven through the following proof. Primarily, (Equation 3) can be equivalently converted into


(10) $$\begin{array}{l} L\ddot{q}+\overset{\cdot }{L}\overset{\cdot }{q}-{\ddot{r}}_{\text{d}}=\ddot{r}-{\ddot{r}}_{\text{d}}=-\beta \left(\overset{\cdot }{r}-{\overset{\cdot }{r}}_{\text{d}}\right)-\zeta \left(r-r_{\text{d}}\right). \end{array}$$

Assuming the position error as ***u*** = ***r*** − ***r***_d_, the above equation is reorganized as $\ddot{u}+\beta \overset{\cdot }{u}+\zeta u=0$, which belongs to a second-order differential equation system. The roots of the corresponding characteristic equation are $s_{1}=\left(-\beta +\sqrt{\beta^{2}-4\zeta }\right)/2$ and $s_{2}=\left(-\beta -\sqrt{\beta^{2}-4\zeta }\right)/2$. Furthermore, According to Equations 8–10, we can analyze that the convergence of this system can be categorized into the three cases (Jin et al., 2017).

- Case i: When β^2^ − 4ζ > 0, we obtain that *s*_1_ < 0 and *s*_2_ < 0 are real numbers with *s*_1_ ≠ *s*_2_. Then, the solution satisfies ***u***(*t*) = ***c***_1_exp(*s*_1_*t*) + ***c***_2_exp(*s*_2_*t*) with $c_{1}\in {\mathbb{R}}^{b}$ and $c_{1}\in {\mathbb{R}}^{b}$ being coefficient vectors determined by the initial state of the system.
- Case ii: When β^2^ − 4ζ = 0, the system has two equivalent characteristic roots with *s*_1_ = *s*_2_ < 0. Therefore, the solution can be deduced as ***u***(*t*) = (***c***_1_ + ***c***_2_)exp(*s*_1_*t*).
- Case iii: When β^2^ − 4ζ < 0, *s*_1_ = *z* + *iy* and *s*_2_ = *z* − *iy* are conjugate complex numbers with *z* < 0. As a result, the solution can be deduced as *u*(*t*) = exp(*zt*)(***c***_1_cos*yt*+***c***_2_sin*yt*).

These cases demonstrate that the position error *u* generated by Equation 3 exponentially converges to a zero vector from any initial states. In other words, it is concluded that Equation 7b enables trajectory tracking control of the manipulator with the unknown mass matrix. The proof is thus completed.

## 5 Simulative results and comparisons

**Figure 2:**
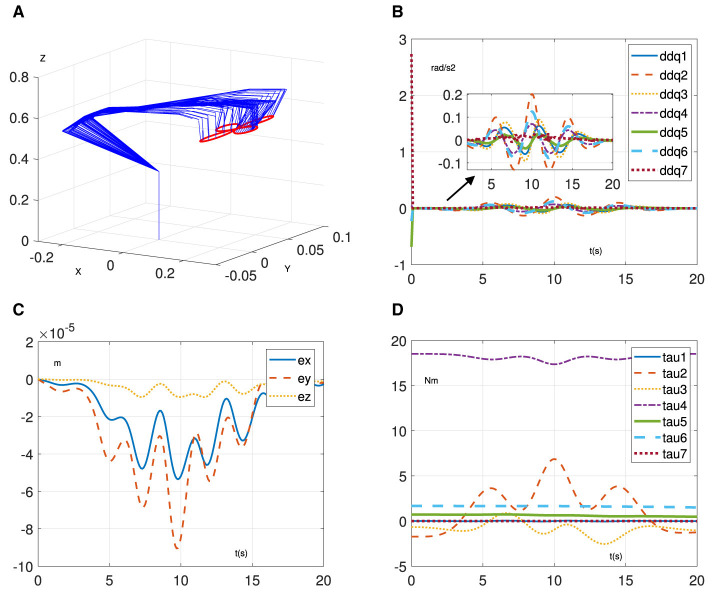
Simulative results of RNN (Equation 7) for trajectory-tracking task on Franka Emika Panda manipulator. **(A)** Motion process. **(B)** Joint acceleration. **(C)** Position error. **(D)** Joint torque.

**Figure 3:**
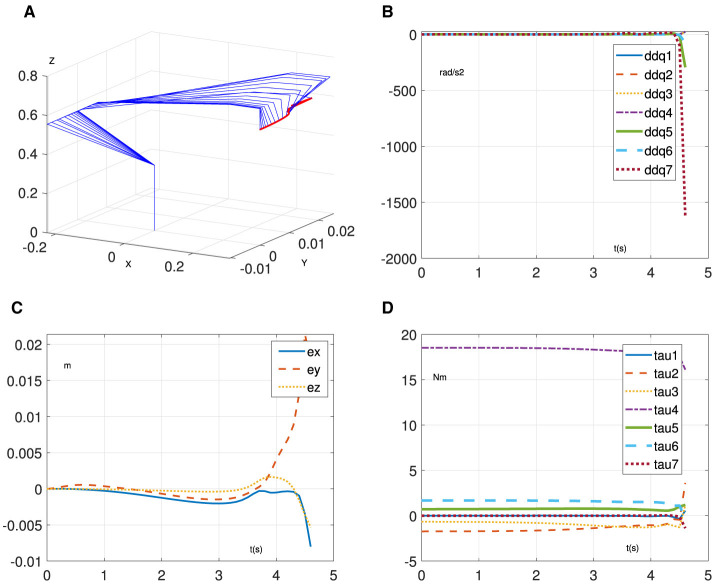
Simulative results of Equation 7b without Equation 7a for trajectory-tracking task on Franka Emika Panda manipulator. **(A)** Motion process. **(B)** Joint acceleration. **(C)** Position error. **(D)** Joint torque.

This section provides simulation experiments to demonstrate the learning and control performance of RNN (Equation 7). Specifically, we test it on a 7-degree-of-freedom manipulator called Franka Emika Panda (Liu and Shang, 2024) to task a four-leaf clover path with α = 10^4^, β = 1, and ζ = 5. In addition, we assume that the mass matrix is unknown and design a random noise matrix with elements < 0.5 to represent its uncertainties. The related results are shown in Figures 2, 3. Figure 2A demonstrates the effectiveness of the proposed method in enabling the manipulator to accomplish trajectory-tracking tasks, even in the presence of an unknown mass matrix. In addition, the initial joint acceleration in Figure 2B is relatively large due to the initial mass matrix error and becomes smooth and normal. Furthermore, the position error keeps the order of 10^−5^ m in Figure 2C. Similarly, in Figure 2D, it can be observed that the joint torque exhibits reasonable variations. However, when the estimation Equation 7b is not considered, achieving the trajectory tracking task based on Equation 7b becomes challenging due to the presence of the unknown mass matrix. As shown in Figure 3A, the manipulator driven by Equation 7a cannot complete the tracking task. Evidently, the joint acceleration became uncontrollable at ~4.5 s, as shown in Figure 3B, and the manipulator system is no longer operational. Furthermore, the position error exhibits divergence in Figure 3C. Similarly, the joint torque in Figure 3D is out of control at 4.5 s. Through the above results, the learning and control ability of the proposed method are verified.

**Figure 4:**
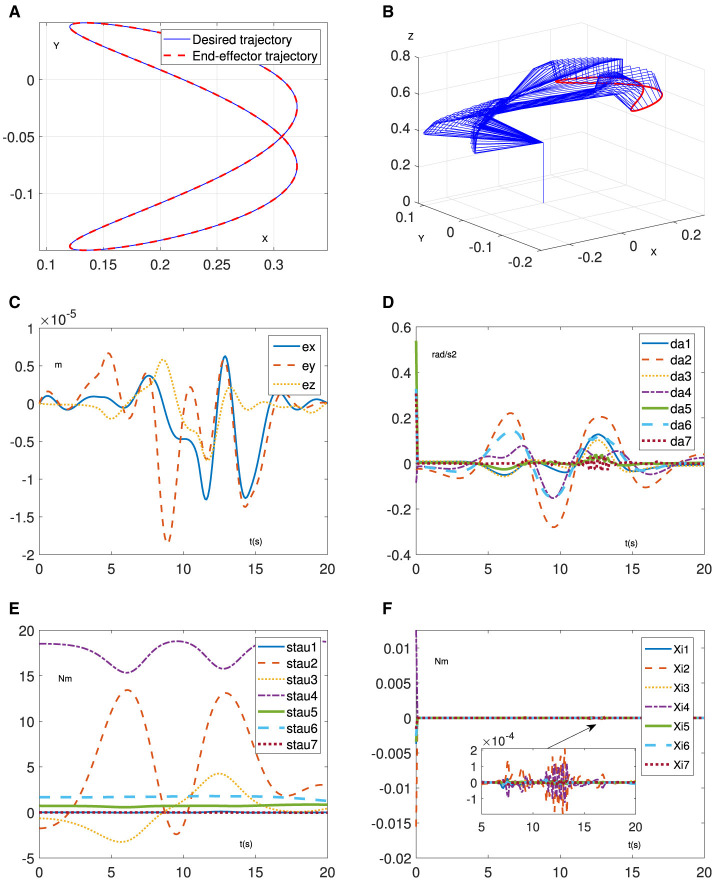
Simulative results of RNN (Equation 7) for trajectory-tracking task on Franka Emika Panda manipulator. **(A)** Desired trajectory and end-effector trajectory. **(B)** Motion process of manipulator. **(C)** Position error. **(D)** Joint acceleration. **(E)** Estimated joint torque. **(F)** Joint torque error with $\xi =\tau -\bar{\tau }$.

To further demonstrate the feasibility of the proposed method, we additionally apply the proposed RNN (Equation 7) to control the Franka Emika Panda manipulator performing a Lissajous trajectory-tracking task. It is noteworthy that the parameters involved are identical to the previous simulation, except for the trajectory-tracking task. The specific results are presented in Figure 4. Specifically, Figures 4A, B demonstrate that the manipulator successfully executes the given trajectory tracking task, taking into account dynamics uncertainties. Furthermore, the positional error, as shown in Figure 4C, is maintained at the order of 10^−5^ m. Additionally, the joint acceleration exhibits normal variations, as shown in Figure 4D. In addition, Figures 4E, F illustrate that the proposed method is capable of compensating for the dynamics uncertainties, with tiny estimated errors of the joint torque. The aforementioned results indicate the effectiveness of the proposed RNN (Equation 7).

In addition, the advantages of the proposed RNN are shown in Table 1, compared with the existing methods. One notable advantage of the proposed RNN (Equation 7) is its simultaneous consideration of both the kinematic and dynamic models. This approach enables the realization of online estimation of the mass matrix and synchronous trajectory tracking.

**Table 1:** Comparisons among different methods for controlling manipulator.

**Different**	**Dynamic**	**Unknown**	**Trajectory**	**Mass matrix**
**methods**	**control**	**issue**	**tracking**	**Online estimation**
RNN (Equation 7)	Yes	Yes	Yes	Yes
Guo et al. (2018)	No	No	Yes	No
Jin et al. (2023)	No	No	Yes	No
Ma et al. (2023b)	No	No	Yes	No
Jin et al. (2017)	No	No	Yes	No
Liu and Shang (2024)	No	No	Yes	No
Shojaei et al. (2021)	Yes	Yes	No	No
Zong and Emami (2021)	Yes	No	No	No

## 6 Conclusion

In this study, we have proposed a recurrent neural network (RNN) to address the challenges of trajectory tracking in manipulator systems with unknown mass matrices. The key idea of our proposed RNN is to establish a connection between the kinematics and dynamics models using joint acceleration signals, considering the motion characteristics and dynamic behavior of manipulators. Primarily, it has incorporated a kinematic controller to generate the desired joint acceleration based on the given task. On this basis, the robot dynamics model and a mass matrix estimator have been designed and integrated into the RNN to enable trajectory tracking in the presence of an unknown mass matrix. Subsequently, theoretical analysis has demonstrated the learning and control capabilities of the RNN. Through simulation experiments and comparisons, we have validated the effectiveness and superiority of the proposed RNN for trajectory tracking control of the manipulator with unknown mass matrix.

In addition to the robot's mass matrix, other dynamic parameters of the manipulator, such as the gravity vector, may also change. In addition, joint constraints help to improve the safety of robot operation. Therefore, future research will focus on estimating multiple dynamic parameters and considering multiple levels of joint constraints.

## Data availability statement

The raw data supporting the conclusions of this article will be made available by the authors, without undue reservation.

## Author contributions

JL: Funding acquisition, Investigation, Methodology, Project administration, Writing – review & editing, Supervision. JS: Data curation, Writing – original draft. HF: Methodology, Supervision, Writing – review & editing. WY: Methodology, Writing – review & editing. XM: Methodology, Writing – review & editing. ZL: Data curation, Methodology, Writing – review & editing.
